# Supergenes are not necessary to explain the maintenance of complex alternative phenotypes

**DOI:** 10.1098/rspb.2024.1715

**Published:** 2024-10-16

**Authors:** Sarah P. Flanagan, Suzanne H. Alonzo

**Affiliations:** ^1^ School of Biological Sciences, University of Canterbury, Christchurch, New Zealand; ^2^ Department of Ecology and Evolutionary Biology, University of California Santa Cruz, Santa Cruz, CA, USA

**Keywords:** genome-wide genetic diversity, alternative mating tactics, frequency-dependent selection, supergene, alternative reproductive tactics, genetic architecture

## Abstract

Evolutionary biology aims to explain the diversity seen in nature. Evolutionary theory provides frameworks to understand how simple polymorphisms or continuous variation are maintained, but phenotypes inherited as discrete suites of quantitative traits are difficult to fit into this framework. Supergenes have been proposed as a solution to this problem—if causal genes are co-located, they can be inherited as if a single gene, thus bridging the gap between simple polymorphisms and continuous traits. We develop models to ask: how are critical supergenes for maintaining phenotypic diversity? In our simplest model, without explicit genetic architectures, three alternative reproductive morphs are maintained in many of the parameter combinations we evaluated. For these same parameter values, models with demographic stochasticity, recombination and mutation (but without explicit genetic architecture) maintained only two of these three morphs, with stochasticity determining which morphs persisted. With explicit genetic architectures, regardless of whether causal loci were co-located in a supergene or distributed randomly, this stochasticity in which morphs are maintained was reduced. Even when phenotypic variation was lost, genetic diversity was maintained. Altogether, categorical traits with polygenic bases exhibited similar evolutionary dynamics to those determined by supergenes. Our work suggests that supergenes are not the only answer to the puzzle of how discrete polygenic phenotypic variation is maintained.

## 1. Introduction

A wide variety of animals exhibit sex-specific morphs that are generally associated with different discrete ways of maximizing reproductive success, which are often referred to as alternative reproductive tactics [1]. These stable polymorphisms are typically maintained in populations through negative frequency-dependent selection (e.g. female morphs in damselflies [2]; Gouldian finches [3]), the interaction of frequency-dependent and density-dependent factors (e.g. side-blotched lizards [4]) and fluctuating selection pressures enabling the partitioning of reproductive success over time (e.g. the feminized dwarf spider morph mates earlier, whereas the alternative morph is able to mate with previously mated females later in the season [5]). When these tactics are fixed within an individual throughout their lifetime, an individual’s tactic often has a large genetic component (e.g. dwarf spiders, side-blotched lizards, ruffs; examples reviewed in [1,6]). The maintenance of fixed tactics has been modelled using explicitly or implicitly simple genetic architectures of one or two genetic loci [4,7–13]. Given that morphs are suites of traits inherited together, some authors have used verbal models to explore the importance of correlational selection and its associated formation of genetic correlations and linkage disequilibrium (LD) across loci [14]. While these relatively simple genetic models do capture the evolutionary dynamics of some empirical examples [7,15], genomic data have accumulated for a number of species suggesting that categorical traits might in fact be underpinned by numerous quantitative genetic loci [16,17].

Consistent with other work suggesting that traits critical to reproductive fitness are determined by the additive contributions of many loci throughout the genome [17,18], several alternative reproductive tactics are associated with many genetic variants throughout the genome which contribute primarily additively to the phenotypes [16]—implying that although the tactics might be discrete and inherited in an apparently Mendelian fashion, their genetic basis is much more complex and multivariate. A particularly intriguing result is that structural variants have aggregated multiple variants contributing to the tactics into a ‘supergene’ (a tightly linked set of genes, often with reduced recombination due to inversions [19,20]) in at least three species with alternative reproductive tactics: the ruff [21,22], white-throated sparrow [23] and dwarf spiders [5]. Supergenes have also been linked to sex-specific traits important to reproduction such as sperm morphology [24,25], head colour [26] and sex-specific migratory behaviours [27]. The potential for supergenes to underpin sexually dimorphic polyphenisms is intriguing, as it could explain the apparent mismatch between simple single-locus population genetic models and the fact that these reproductive tactics are suites of complex traits [7]. Supergenes, with their reduced recombination between variants, also provide a mechanism for individuals with largely similar genomic information to harbour fixed genetic variation without requiring differential gene expression. Examples where supergenes apparently underpin sexually dimorphic phenotypes tend to involve multiple morphs of one sex, usually males. All known examples of alternative morphs determined by supergenes include a female-mimic morph, including ruffs [21,22], dwarf spiders [5] and white-throated sparrows [23]. In the ruffs, a third male morph exists, with an intermediate phenotype between the dominant or ‘classic’ male morph and the female mimic [21,22]. In some cases, a heterozygous genotype is lethal (e.g. ruffs [21,22]), but not in others (e.g. dwarf spiders [5]). In the white-throated sparrow and the ruff, these structural variants are successful in coding for these variable phenotypes because they include androgen receptors [28]—the white-throated sparrow supergene contains the oestrogen receptor gene which directly influences the aggressive aspects of the dominant male phenotype [29]. Despite the potential promise of supergenes to explain the existence of genetically determined alternative reproductive tactics, the existence of these inversion polymorphisms has not been studied in the majority of species with fixed alternative tactics.

Thus, an open question is: to what extent do supergenes facilitate the maintenance of multiple morphs versus the evolution of multiple morphs facilitating the evolution of supergenes? This question is difficult to address empirically but is important for understanding the genetic mechanisms underpinning adaptive genetic variation. For example, if a supergene exists and a mutation arises within it that affects the morph traits, the genetic variation within the supergene itself could have eroded due to relaxation of selection and reduced recombination. As such, only the mutation would be likely to show significant genetic variation—this could make a supergene with functional effects difficult to detect empirically. This is especially true in cases such as the white-throated sparrow, in which the gene with a major effect is an oestrogen receptor [29], which probably affects the expression of many genes. Furthermore, it is currently unknown to what extent genetic architectures—and the constraints inherit to them—impact the dynamics of the frequency- and density-dependent mechanisms that maintain simple cases of genetic variation in populations.

An additional challenge arises from limitations in the empirical data that can be used to identify genetic architectures of these traits and uncover the mechanisms governing their evolution. The majority of examples involved researchers first generating a high-quality reference genome and subsequently performing whole genome resequencing of individuals of each morph and sex to identify variants within populations that are associated with the phenotypic tactics [5,16,21–24,29,30]. Alternative approaches involve linkage mapping [31] or the investigation of differential gene expression between morphs [32–34]. These methods have proven successful in some cases, although for many other types of traits the ability to detect causal variants is generally limited [35–37]. Regardless, despite the potential ability to uncover the most important evolutionary processes in the evolution of these traits using genomic data [38–40], the power might be substantially limited in real-world samples [41,42]. In part, this limitation is due to lacking a predictive understanding of how different genetic architectures can influence evolutionary dynamics of polygenic traits.

To tackle these problems, we use models to test how different genetic architectures affect the maintenance of multiple male reproductive morphs. We focus on first identifying key reproductive parameters that influence the maintenance or loss of morphs using an analytical haploid model. Using a simulation model, we test whether different types of diploid genetic architectures (single locus, polygenic and polygenic supergene) change the presence of and relative frequencies of morphs that are maintained. We then use the output of these simulation models to test the accuracy and ability of current empirical methods to identify the causal genetic variants underlying the alternative reproductive tactics.

## 2. Methods

Our model considers a biological scenario in which males can display to attract females and provide obligate male-only parental care. To allow us to answer our questions about supergenes facilitating multiple morphs, we consider two independent traits, courtship and parental care, both of which are expressed only in males. We assume that all females in the population prefer males with the courting trait, but females do not know when choosing a male whether that male will also provide care. Males who do not court can only reproduce by sneaking, and males who display but do not provide any parental care will not produce viable offspring. Thus, our model has four potential tactics: courter/parents (CP); courter/non-parents (CN); non-courters/parents (NP); or non-courters/non-parents (NN). Our model assumes non-overlapping generations and no environmental effects on traits. While this model does not directly apply to all examples of alternative reproductive tactics, it is biologically reasonable and common even if not universally true. We expect that this model can be generalized to species in which one sex (but not the other) has two fixed traits that affect mating success and the survival of their offspring.

To test the importance of genetic architecture in male morphs, we implemented three models. First, we developed an analytical model that reflects haploid inheritance of a single locus that encodes morphs. This model did not include recombination or mutation. The goal of this model was to determine parameter settings that lead to the persistence of three morphs versus the fixation of a single morph in the population. Second, we used a stochastic individual-based model with two diploid loci, one for each trait (courting and parenting), that incorporated recombination, mutation and demographic stochasticity. Finally, we expanded the stochastic model to incorporate polygenic inheritance of the two traits, with two different explicit physical genetic architectures: quantitative trait loci (QTLs) distributed at random throughout a genome including non-coding loci and a supergene architecture, where QTLs were co-located in a non-recombining region of a single chromosome. We assumed all QTLs were autosomal. Each allele at a QTL was assigned a randomly drawn phenotypic effect, and an individual’s quantitative phenotypic value was determined by the sum of all their allelic effects (i.e. traits were entirely additive). In these additive, polygenic models, male traits were determined by a threshold function. The value of the threshold was constant for each iteration of the model and was initialized as the mean phenotypic value at generation 0, prior to any model iterations. In all cases of the individual-based models, sex was assigned instead by a draw from a binomial distribution with a probability of 0.5 of being female.

**Figure 1.**
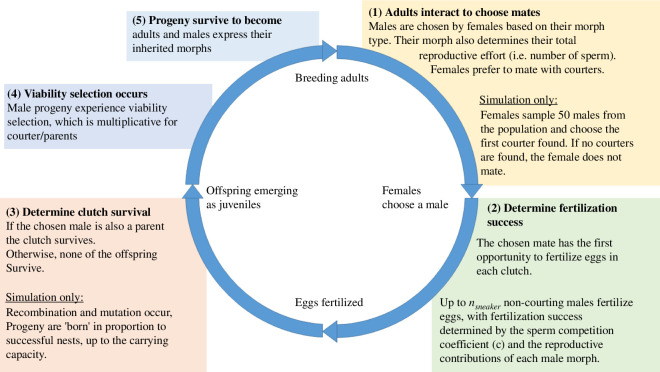
Diagram of the biological life cycle modelled in both the analytical model and the simulation model. The model has five phases, starting with (1) Adults interact to find mates. Differences between the models are articulated in the diagram.

In all three models, each generation has five distinct phases: (1) mate choice; (2) fertilization; (3) parental care; (4) viability selection; and (5) maturation to adulthood (figure 1). We will describe each of these phases and highlight differences between the models when appropriate.

### (a) Stage 1: mate choice

In the mate choice phase of the model, we assumed females always preferred to mate with males displaying the courtship trait and did not mate with non-courting males. If a female decided to mate with a male, he received all of her eggs to care for.

In the analytical model, this mate choice phase determined to total number of clutches in the population, n . The general calculation for the number of clutches is


$$n=w_{s}\left(N_{m}f_{CP}+N_{m}f_{CN}\right)+\left(1-w_{s}\right)\left(N_{m}f_{NP}+N_{m}f_{NN}\right),$$

where $w_{s}$ specified the probability a female chose a courting male (CP or CN), $N_{m}$ was the number of males in the population, and the f terms represented the frequency of each morph. To match our model assumptions, $w_{s}=1$ , so n simplified to $w_{s}\left(N_{m}f_{CP}+N_{m}f_{CN}\right)$ .

The simulation model incorporated stochasticity at this stage of the model. Females searched through a set number of males and chose the first attractive male (i.e. the first courting male she encountered). If a female did not find an acceptable mate, she did not mate.

### (b) Stage 2: fertilization

A chosen male was not guaranteed paternity of an entire brood, because non-courting males were able to fertilize a proportion of the eggs. The chosen parent had first-male fertilization advantage. The consequence of this scenario is that the only mechanism by which non-courters can produce offspring is through sneak fertilizations. Specifically, we modelled sneaking by specifying a total number of sneakers, $n_{sneak}$ , that could fertilize via sneaking in each clutch, and each sneaker could contribute up to their individual reproductive allocation (where $r_{NN}=r_{NP}$ ) offset by the sperm competition coefficient ( c ). We explored the effect of different values of these parameters in the analytical model, but for all simulation models analysed here, $n_{sneak}=3$ and c=0.5 . We also assumed inherent ‘reproductive allocations’, $r_{M}$ (where M designated the morph and contained {CP,CN,NP,NN} ), for each morph, which sets a ceiling on the number of offspring any male can sire—this is analogous to sperm quantity, which frequently is higher in sneakers than dominant morphs in empirical examples [43].

In the analytical model, we modelled the fertilization success for morphs by considering their fertilization success in clutches laid by the females who chose them, $e_{o_{M}}=f_{M}N_{m}r_{M}w_{s_{M}}$ (which will only be non-zero for courting morphs). We specified the maximum number of possible fertilizations arising from sneaking for a given non-courting morph was $N_{s}=ncn_{sneakers}r_{NN}$ . If the number of fertilizations arising from sneaking was smaller than the morph’s potential population-level reproductive effort (i.e. if $N_{s}<f_{M}N_{m}r_{M}$ ), the number of eggs fertilized through sneaking ( $e_{s_{M}}$ ) for that morph was the difference between $N_{s}$ and $f_{M}N_{m}r_{M}$ , truncated at zero. Otherwise, the number of eggs fertilized through sneaking was $f_{M}N_{m}r_{M}$ .

In the simulation model, we incorporated recombination and mutation during the fertilization process. Recombination occurred independently on chromosomes inherited from the mother and the father. The number of recombination events for a given chromosome was determined by drawing from a Poisson distribution with the recombination rate as the mean, which we set to 0.2. For each recombination event, breakpoints were chosen by randomly drawing from the total number of markers on a chromosome. These breakpoints defined the segments of the chromosome that experienced recombination, and each segment was randomly chosen to be assigned the genotypes and effects from the parents’ maternal or paternal chromosomes. When the genetic architecture was that of a supergene, if the breakpoints fell within the region containing the aggregated QTLs for the traits, the offspring was considered not viable, mimicking suppressed recombination as observed in empirical examples (e.g. ruffs [21,22]).

After recombination, mutation occurred at a rate of μ at each locus. For a given individual, the number of mutations introduced was determined as $2\mu n_{SNPs}n_{chrom}$ , where $n_{SNPs}$ was the number of single nucleotide polymorphisms (SNPs) and $n_{chrom}$ was the number of chromosomes in the simulation. The location of each mutation was randomly selected and the allele at that location was randomly chosen from the set of existing alleles. The mutation also affected the phenotypic effects of QTLs by altering the original effect by a value drawn from a normal distribution with a mean of 0 and a standard deviation of $\sigma_{\mu }$ . Finally, offspring were also randomly assigned a sex.

### (c) Stage 3: parental care

We assumed for simplicity that males with the parental care trait have 100% offspring survival while males without the parental care trait have 0% survival, independent of the male’s courtship trait. This was straightforward to model in the simulations: after the males were assigned their paternity shares (during fertilization), the clutch survived if the chosen male was also a parent. Because of this assumption, and those in Stage 1, the courter/non-parent morph never produced viable offspring. However, this morph was included in the models at the initial stages because the two traits were set up to be inherited independently in the initial conditions for each run of the model.

In the baseline model, we modelled this by defining the selection coefficient for clutch survival, $w_{n_{M}}$ , which determined the success of each morph’s clutch, assuming the morph was chosen by a female: $o_{o_{M}}=e_{o_{M}}w_{n_{M}}$ . To match our model assumptions, $w_{n_{CP}}=w_{n_{NP}}=1$ and $w_{n_{CN}}=w_{n_{NN}}=0$ . We also calculated the number of surviving offspring from sneaking as


$$o_{s_{M}}=e_{s_{M}}\frac{\Sigma o_{o_{M}}}{\Sigma e_{o_{M}}}.$$

Because we assumed obligate parental care, the fraction was either 1 or 0, depending on whether the morph associated with the clutch had the parental care trait. Males with the parental care trait were assumed to care for all offspring in the clutch, including those arising from sneaking. Each morph’s total number of offspring produced ( $o_{M}$ ) was the sum of $o_{o_{M}}$ and $o_{s_{M}}$ .

### (d) Stage 4: viability selection

We assumed viability selection only affects male offspring, as a cost imposed on displaying courting and/or parental care (to reflect empirical patterns of fitness costs to male displays [44] and care [45]). In the baseline model, we multiplied the viability selection coefficient ( ${w_{v}}_{M}$ ) by the surviving number of offspring per morph ( $o_{M}$ ), to determine the number of surviving juveniles per morph: $j_{M}=o_{M}w_{v_{M}}$ . In the simulation model, we calculated a survival probability for each male offspring, as $e^{\frac{-\left(morph-\theta \right)\left(morph-\theta \right)}{2\omega_{v}}}$ , where $\omega_{v}$ represents the strength of viability selection, morph is 1 for courters and parents and 0 for non-courters and non-parents, and θ=0 (i.e. selection favours the non-courters and non-parents). For each individual, a random value between 0 and 1 was drawn from a uniform distribution; if the number was larger than his survival probability, that individual did not survive to adulthood. In both models, viability selection acted multiplicatively on courter/parents.

### (e) Stage 5: maturation to adulthood

In the baseline model, the final stage in the life cycle was calculating the frequency of each morph in the next generation by dividing the surviving number of juveniles per morph after viability selection ( $j_{M}$ ) by the sum of all surviving juveniles: $f_{M}=\frac{j_{M}}{\Sigma_{i=1}^{4}j_{i}}$ .

In the simulation model, the total number of surviving offspring in the population depended on the proportion of population reproductive success achieved by each individual male and female (i.e. each generation, the number of offspring created was equal to the carrying capacity). The carrying capacity was arbitrarily set as 1000 to constrain the maximum population size. If the number of surviving offspring was larger than the carrying capacity, offspring were randomly selected to become adults (i.e. independent of their morph).

### (f) Evaluating model outcomes

#### (i) Baseline analytical model

To evaluate the outcomes of this analytical model, we iterated forward in time for 100 generations from 1659 combinations of initial morph frequencies and repeated those iterations across five values for the sperm competition coefficient ( c = {0..1}), 21 values for the relative reproductive coefficient ( $r_{M}$ = {0…2}), and three values of the number of sneakers ( $n_{sneakers}$ ={1..3}). The iterations were implemented in R v. 4.1.1 [46] using some features of tidyR [47]. We also ran a subset of parameter combinations for 1000 generations for comparison with the simulation model (more details below).

We were interested in identifying the regions of parameter space that resulted in the largest diversity of male morphs. We leveraged existing ecological mathematical tools to summarize diversity of a system with a single metric—specifically, Shannon’s diversity index. We calculated this index in the 100th generation using the R package vegan [48] and subsequently visualized the diversity of the population using contour plots produced by plotly [49] in a shiny app that relies on R packages shiny [50], shinydashboard [51], rsconnect [52], dplyr [53] and rmarkdown [54–56]. The interactive app is available at https://spflanagan.shinyapps.io/morph_predictions/ (see electronic supplementary material, figure S1 for a static image).

This model provided baseline predictions for parameter values likely to facilitate morph diversity within a population against which to evaluate the effect of more complex and explicit genetic architectures.

#### (ii) Individual-based simulation model

We initialized four replicate populations with identical starting conditions to evaluate the effect of stochasticity on the simulation models. These identical conditions included randomly generated values for the allelic effects of QTLs, the exact number of individuals of each sex and morph, and the thresholds for determining morph traits (i.e. the values at which an individual is deemed a courter versus a non-courter and a parent versus a non-parent). For each set of four populations, we ran 10 000 generations followed by 2000 ‘experimental’ generations. Doing so ensured that the population reached a steady state in which we could examine any stable polymorphisms or cyclical fluctuations in morph frequencies. We ran at least three sets of these identical replicates for all parameter combinations.

To explore the effects of genetic architecture on morph maintenance, we used two sets of demographic and reproductive parameters that were identified by the baseline model as either facilitating high or low morph diversity. Within each of these parameter settings, we compared model outcomes with for cases with each combination of 2, 4 or 8 chromosomes and 8, 16, 32 or 64 QTLs underlying each trait. We also tested three supergene sizes, which are similar to empirical supergene examples: occupying 5% of a chromosome (similar to the spider *Oedothorax gibbosus* [5]), 25% of a chromosome (similar to the ruff [21,22,57]), or 50% of a chromosome (similar to rainbow trout [27] and white-throated sparrow [58]). In all cases, a chromosome comprised 1000 loci (including marker loci and QTLs).

#### Analysing multilocus genotypes

To provide a link between the predictions of our theoretical models and empirical data, we analysed the genetic diversity of the populations at the final generation of the individual-based simulations. This is an equivalent analysis to the resequencing conducted in empirical studies [5,16,21–24,29,30]. The general idea behind these genome-wide analyses is that QTLs will have higher genetic diversity than background marker loci because balancing selection is favouring multiple alleles at those loci, with genetic variation existing between the morphs and specific variants associated with the different phenotypes.

To implement this analysis, we used vcfR [59] to estimate observed heterozygosity (i.e. a measure of variation at a locus) for all markers in the genome, including QTLs, and compared the frequencies of alleles between morphs using $G_{ST}$ , which is a measure between 0 and 1 that indicates how different the frequencies are between groups. Additionally, an association between male phenotypes and genetic markers was tested for using GWASpoly [60]. Finally, we used vcftools [61] to test for signals of balancing selection at the QTL loci compared with non-QTL loci by calculating the Tajima’s D statistic. Because linkage cause loci near to the actual target of selection (the QTL) to have elevated variation as well, we identified ‘peaks’ of differentiation ( $G_{ST}$ ) and association (GWAS *p*-values) and determined the proportion of these peaks that were within a 50 basepair window of one of the true QTLs.

We evaluated whether QTLs evolved LD due to shared selection pressures, and not only physical linkage by estimating LD between all loci using vcftools [61]. We then estimated summary statistics for each locus and compared LD between QTLs to LD among non-QTLs. Because we had far fewer QTLs than non-QTL loci in all of the iterations, we also used resampling of the non-QTLs to estimate the average LD, using 999 resampled estimates with the actual number of QTLs in each replicate. This procedure was applied to the genotype data from the final generation of all simulations with genetic architectures.

## 3. Results

### (a) Analytical model reveals many regions of parameter space where multiple morphs are maintained

**Figure 2.**
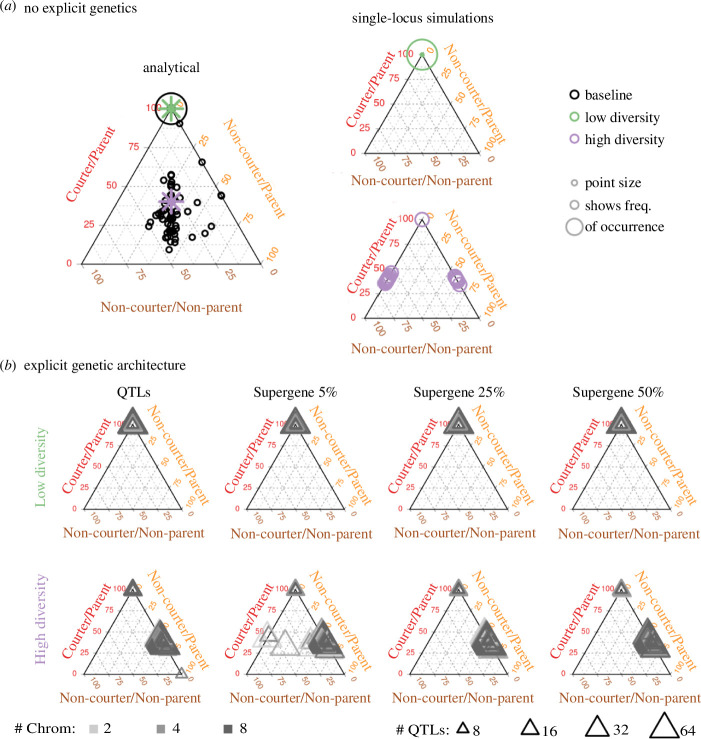
The inclusion of recombination and stochasticity alter the frequency of male morphs and explicit genetic architectures affect which morphs are maintained under balancing selection. In all diagrams, each point in the triangle represents a different combination of the predicted frequency of the three male morphs, where the vertices represent 100% of each morph and each edge represents relative percentages of two out of the three morphs. (*a*) A comparison of the baseline analytical model and the architecture-free single-locus simulations. In these diagrams, point size reflects the number of parameter settings resulting in that exact combination of morph frequencies. The left panel represents the predictions of the baseline model, in which most parameter combinations result in the fixation of the courter/parent morph but many retain three morphs. The outcomes of the chosen low-diversity and high-diversity parameter sets are marked with a green and a purple asterisk, respectively. On the right are the outputs from the individual-based simulation models without explicit genetic architectures for the chosen low-diversity parameters (top) and high-diversity parameters (bottom). Most low-diversity replicates result in fixation of the courter/parent, with some having one or two non-courter/parent individuals in the final population, while the high-diversity replicates include maintenance of two morphs (courter/parent and non-courter/parent or courter/parent and non-courter/non-parent) and the fixation of the courter/parent morph. (*b*) Adding explicit genetic architectures affects which morphs are maintained for the high-diversity parameters, but the number of QTLs or chromosomes are not driving factors, and low-diversity parameter settings resulted in the predicted outcomes. The ternary diagrams show the final frequencies of the morphs from individual-based simulations with QTLs distributed throughout the genome (left column) or clustered into supergenes that make up 5, 25 or 50% of the chromosome the supergene is situated on. Colours represent the number of chromosomes considered by the model and the size of the points depicts the number of QTLs underlying each trait. In low-diversity scenarios, regardless of genetic architecture, the courter-parent morph is the one that is retained, same as in the baseline model and architecture-free simulations in (*a*). Unlike in the baseline and architecture-free models, in high-diversity scenarios, the non-courter/non-parent individuals are rarely retained and are only retained in a few cases when supergenes are very tightly linked.

The purpose of exploring the baseline analytical model was to identify the parameter space in which, in a genetically simple model in which the genetic architecture is not explicit, more than one male type is predicted to persist, thereby creating a baseline for comparison with the simulation results. The courter/non-parent morph was never maintained in the population. This result makes intuitive sense, because if a female chooses to mate with a courter/non-parent, the offspring will always die (given our parameter settings) (figure 2*a*).

To quantitatively identify regions of parameter space that resulted in multiple morphs, we focused on parameters yielding Shannon–Wiener diversity indices greater than or equal to 1 . The regions of the parameter space that have positive diversity values correspond to scenarios where three of the four morphs were retained (courter/parents, non-courter/parents and non-courter/non-parents), although the dominant morph depended on the specific parameter combinations. With equal starting frequencies, after 10 000 generations, 18.82% of parameter combinations that retained polymorphism resulted in the courter-parent morph comprising greater than or equal to 50% of the population. The remaining 81.18% of parameter combinations resulting in the maintenance of alternative morphs had more equal representation of all three morphs, with the average proportion of the population being 30.6% ( ± 1.29%) courter/parents, 34.78% ( ± 0.75%) non-courter/parents and 34.62% ( ± 0.68%) non-courter/non-parents (figure 2*a*, electronic supplementary material, S1).

Overall, the parameters affecting whether diversity was maintained after both 100 and 1000 generations were the initial frequencies of the morphs, sperm competition ( c ), relative reproductive allocation ( r ) and the number of sneakers allowed ( $n_{sneak}$ ). Some initial starting conditions took more than 100 generations to reach a consistent (i.e. equilibrial) state, but the contributions of the initial frequencies and these three parameters remained qualitatively consistent between 100 and 1000 generations.

As expected, larger sperm competition coefficients ( c ) resulted in a higher likelihood of non-courters being retained in the population, which makes sense given that c affects the proportion of eggs within a clutch that a non-courting male can successfully fertilize. Sperm competition interacted with the reproductive allocation ratio, r , such that when c>0.2 , the maximum diversity was achieved when 0.5≤r≤1 . As an illustrative example, if r=0.5 , c=0.2 , and the non-courting male’s number of eggs he can fertilize is 8, the non-courting male would fertilize $\frac{\left(8\right)\left(2\right)}{\left(8\right)\left(0.5\right)+\left(2\right)\left(0.2\right)\left(8\right)}$ = 0.222 of the offspring. The effects of sperm competition on morph diversity were further influenced by $n_{sneak}$ , with larger contributions from an individual non-courting male (e.g. larger c ) being required to maintain diversity of morphs when fewer sneakers were allowed (electronic supplementary material, figure S1). This result makes sense because the number of sneakers is multiplied by the sperm competition coefficient to determine the proportion of offspring fertilized by the non-courters for a given clutch. These analyses therefore helped to build an understanding of how the different parameters affected model predictions and provided a baseline against which to compare our simulation results.

We used these results of the baseline model to select parameter values for the individual-based simulation model. We selected one set of parameters predicted to produce low-diversity populations (i.e. those where only one morph was retained) and another set expected to produce high-diversity populations (i.e. where 2 or more morphs were retained). The chosen parameter values are shown in table 1.

**Table 1.** Table of parameters selected to create a high-diversity population. All morphs started at equal frequencies.

parameter	low-diversity value	high-diversity value
ratio of reproductive investment, *r* (courter to non-courter ratio)	2.0	0.70
max number of offspring CP males can produce	8.0	6.00
max number of offspring CN males can produce	8.0	6.00
max number of offspring NP males can produce	4.0	8.00
max number of offspring NN males can produce	4.0	8.00
*c* (sperm competition coefficient)	0.5	0.75
number of sneakers	2.0	2.00

### (b) Recombination, mutation and stochasticity result in fewer morphs maintained

A striking difference between the analytical model and the simple Mendelian simulation model is that only two morphs are maintained with parameters that retained three morphs in the analytical model (figure 2*a*; see electronic supplementary material, figure S2 for outcomes with alternative mating dynamics). Furthermore, stochasticity played a major role in which morph coexisted with the courter/parent morph, even when the parameter values used were identical (electronic supplementary material, figure S3). Variation in outcomes among replicate runs can be attributed to the many sources of random chance incorporated in the model: stochasticity associated with finite population sizes (i.e. genetic drift), the randomness of recombination, and the finite mate search process (in which females might not find a suitable mate, even if one exists in the population) all contribute to the within-replicate variation observed. Altogether, including realistic genetic components reduced the total diversity of morphs compared with a deterministic single-gene model.

#### (i) Polygenic architectures reduce effects of stochasticity, with supergenes having little effect on the maintenance of morphs

In the low-diversity parameter space, the outcomes remained consistent with the single-locus model (and the predictions of the mathematical model): courter/parents dominated the populations, with complete fixation of the morph in the majority of runs, and any variation existing in the form of one or two individuals (courter/parent frequencies were greater than or equal to 99% in all cases), which were a result of stochasticity and recombination as opposed to balancing selection favouring multiple morphs (electronic supplementary material, figure S4). These results were consistent across all genetic architectures, including QTLs distributed across two, four and eight chromosomes and QTLs clustered in supergenes spanning from 5 to 50% of a single chromosome. In the high-diversity parameter space, polygenic loci distributed throughout the genome resulted in the maintenance of the courter/parent trait at a frequency of 50.7% ( ± 1.59%), with the non-courter/parent being the dominant morph. These proportions are remarkably consistent across genetic architectures (figure 2*b*). Most simulations with supergenes resulted in very similar final morph frequencies (49.21 ± 0.88%), and only occasionally resulted in either three morphs being retained or the non-courter/non-parent morph replacing the non-courter/parent. The size of the supergene did not substantially affect the final morph frequencies, nor did the total number of QTLs underlying each locus. A consequence of the stabilization of morph frequencies was a reduction in the strength of selection acting in the final generation relative to the first generation of the model (electronic supplementary material, figure S5).

Our initial expectation was that supergenes would follow a pattern more similar to the single locus results, because empirical examples have demonstrated Mendelian inheritance patterns approximating single-locus models (e.g. [21]). To better understand the reasons for the similarity between genome-wide QTLs simulations versus the supergene simulations, we visualized the QTL haplotypes in supergenes (see electronic supplementary material, figures S6 and S7 for representative images). These visualizations suggest that because of the additive, polygenic nature of our traits, individuals have multiple pathways to creating the morphs in terms of genotypes at each QTL, even when QTLs are segregating together in a supergene. This genetic diversity reflects the continuous nature of the traits that additively define a discrete morph, and the fact that threshold traits can harbour hidden diversity. These findings suggest that supergenes are not necessary for maintaining multiple reproductive morphs by all causal loci having the same variants within each morph type.

#### (ii) Genome-wide genetic diversity is maintained even when trait diversity is low

**Figure 3.**
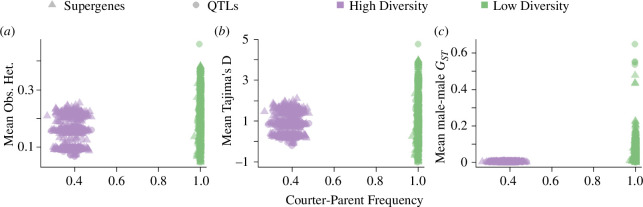
Genome-wide genetic diversity in the final generation of the simulations was not influenced by genetic architecture or the phenotypic diversity (i.e. morph frequencies), but did differ between low- and high-diversity parameter settings. Shown are genome-wide observed heterozygosity (*a*), Tajima’s D (*b*), and male–male $G_{ST}$ (*c*).

Supergenes enable specific combinations of alleles at multiple loci to be inherited together as a single haplotype; as such, we expected that the simulations with supergenes would evolve to have lower within-morph genetic diversity at QTLs (i.e. low observed heterozygosity) than the genome-wide QTLs scenarios. Surprisingly, we found that the average genome-wide expected heterozygosity was not substantially different in simulations with genome-wide QTLs versus QTLs in supergenes in the final generation of the simulations, but differed between low- versus high-diversity parameter settings ( $F_{3,1636}$ = 111.3, *p* < 0.0001; diversity p < 0.0001; architecture p = 0.399; genome-wide mean = 0.15, supergene mean = 0.16; Figure 3*a*; electronic supplementary material, figure S8). In most runs, the heterozygosity at QTLs did not significantly differ from heterozygosity at neutral markers (mean *p*-value from *t*-tests is 0.5 ± 0.01). Tajima’s D, which tests for directional or balancing selection, also reflects genetic variation in a sequence of DNA, did not deviate substantially between genetic architectures and did not relate to final morph frequencies (figure 3*b*; electronic supplementary material, figure S8). Average LD was higher for QTLs in supergenes than randomly distributed QTLs (electronic supplementary material, figure S9).

Similarly, we expected supergenes to show more differentiation between morphs, since their QTLs segregated together. However, $G_{ST}$ between male morphs was also not predicted by the frequency of courter/parent morphs (figure 3*c*; electronic supplementary material, figure S9). Significant peaks in genome-wide $G_{ST}$ estimates were identified, and we evaluated whether QTLs were located near to those $G_{ST}$ peaks (i.e. we identified if $G_{ST}$ outliers were associated with true QTLs). From this, we then calculated the proportion of true QTLs that were located in these significant $G_{ST}$ peaks and found that under high-diversity parameters, on average 63.04 ± 1.09% of randomly distributed QTLs and 74.31 ± 0.9% of QTLs in supergenes were found in $G_{ST}$ peaks, irrespective of the courter/parent frequency (electronic supplementary material, figure S10).

A consequence of this high genetic diversity underpinning the categorical traits appears to be high error rates in detecting true QTLs using genome-wide association tests. As expected, the detection of true QTLs in a GWAS was more likely when fewer QTLs underpin the trait, especially if those QTLs are aggregated on fewer chromosomes (electronic supplementary material, figure S11). The type of genetic architecture (genome-wide QTLs versus supergenes) had a minimal effect on the detection of QTLs. The QTLs for parent traits were more reliably detected by GWAS than the courter QTLs, even though in the majority of simulations the final population only contained parental males (figure 2*b*). The higher probability of detecting parent QTLs could be attributed to stronger selection on that trait (i.e. being a non-parent was more costly than being a non-courter). To summarize, a surprising degree of genetic diversity was retained in our simulations despite fixation of some morphs, which impeded the detection of causal loci.

## 4. Discussion

Understanding how the genetic architecture of phenotypic polymorphisms facilitates or constrains their maintenance and evolution is crucial for understanding the mechanisms shaping diversity. Yet, we do not fully understand how polygenic phenotypes can be inherited as discrete suites of traits. Using a simulation model, we show that the genetic architecture—specifically, the number of loci and the extent of linkage among them—affects the persistence of complex discrete phenotypes. In our single-gene simulation models, under parameter conditions that favoured the maintenance of multiple morphs in simple single-gene simulation models, which male morphs were retained was determined due to stochasticity. However, when more complex architectures are modelled using the same parameters, specific combinations of morphs were retained; thus, genetic complexity of traits reduced the importance of stochasticity and increased the maintenance of phenotypic and genetic diversity. This phenomenon was probably due to genetic correlations between traits and loci. In contrast to expectations, we did not find that supergenes increased the probability that multiple morphs are maintained in the population. QTLs organized into supergenes (in which causal SNPs are physically co-located and experience reduced recombination), although associated with high LD, did not have qualitatively different outcomes in terms of the maintenance of discrete morphs from QTLs distributed genome-wide. In addition, unexpectedly high levels of genetic diversity were retained within morphs in the supergene scenarios.

### (a) Supergenes have similar outcomes to randomly distributed quantitative trait loci

The discovery of supergenes underlying polymorphism was hailed as an explanation for how complex suites of traits could be inherited in a discrete manner, when they must also be polygenic [62]. We provide compelling evidence that additive genetic variation need not be co-located in a non-recombining region of a chromosome for multiple morphs to be maintained. We focus on courting and care phenotypes as two exemplars of polygenic traits, but expect our results would be applicable to other traits involved in alternative tactics, such as aggression. Considering additional forms of complexity in genetic architectures is a fruitful area for future research. For example, the steroid hormone receptor genes in white-throated sparrow and ruff supergenes [28] would be better approximated by a logarithmic distribution, which differs from the additive distribution of allelic effects that we used. Additionally, many polymorphisms could rely on dominance hierarchies (e.g. as modelled by [10]) and could even be underpinned by dominance reversals between morphs [27] or sexes [63], which were not possibilities considered in our model. Regardless, we provide the important result that discrete morphs can be maintained within populations even without discrete genetic architectures with a threshold-based switch, contrary to some previous predictions [64]. Our results indicate that discrete morphs need not have causal genes co-located in a supergene but can still follow the single- or two-locus phenotypic inheritance patterns seen in side-blotched lizards [7] and damselflies [10], and other species with fixed alternative reproductive tactics that do not yet have evidence of supergenes.

### (b) Unexpected genetic variation was maintained even when traits were fixed

Comparing our model predictions with predictions from the literature on sexually antagonistic selection on autosomes can be informative because both scenarios involve balancing selection on shared genomic regions [13]. In our model, balancing selection arises from fitness trade-offs between reproductive investment and survival in males, which creates conflicts among morphs, whereas autosomal sexually antagonistic selection involves conflicts between sexes over similar trade-offs. Unlike models of sex-specific selection, which have found that under conditions resulting in balancing selection on males and females, genetic variation can be maintained [65], but that most genomic regions will not show excessive variation [42], our models predict the maintenance of variation in phenotypes and genotypes. Additionally, we detected high levels of genetic differentiation between male types, even without strong selection, which is opposite to the predictions for sex-specific selection [66]. As such, our model highlights how demographic factors, stochasticity and genomic effects such as pleiotropy and linkage can affect genetic variation and potentially obscure putative genome-wide signals of selection.

Furthermore, we expected the supergenes to evolve to contain single haplotypes for each morph, as has been reported in empirical examples of supergenes controlling complex phenotypes (e.g. [5,21,22,24]). A striking difference in our results from those of the empirical examples is that we do not see fixed alleles exclusively in one morph or the other. Instead, quantitative variation is retained within the supergenes, and we do not observe discrete haplotypes underlying the categorical morphs. If we compare our supergene results with our QTL results, as expected, LD was elevated within the supergene region in our model, and similarly high levels of LD were not observed in the genome-wide QTL models (electronic supplementary material, figure S10). However, measures of genetic variation and between-morph differentiation were similar in the two types of models (figure 3).

These unexpected levels of genetic diversity might have also impaired our ability to detect the causal QTLs using methods such as genome-wide association studies. We only identified less than or equal to 50% of true QTLs, with many false positives. Low true positives and high false positives are consistent with other simulation studies of genome-wide studies of selection and trait associations [42], especially for genome-wide associations with dichotomous traits [37]. In this study, these limitations are consistent with both genetic architectures, so appears to not only be driven by the extreme LD within supergenes that makes associations difficult to identify [24]. Our results suggest that genome-wide association studies alone are likely to miss key causal variants, even if such approaches have been sufficient in identifying causal regions in some species [5,16,21,22,24,26].

### (c) Empirical scenarios likely to fit this model

Like any theory, our model has several assumptions that best capture some empirical scenarios more than others. First, we prevented the courter/non-parent morph from persisting by assuming that those males do not have any reproductive success. We therefore only allowed at most two or three alternative male phenotypes to persist in populations. These are the two most common types of sex-specific, multi-morphic, fixed (i.e. heritable) morphs observed empirically [6]. Our model’s assumptions about morph reproductive success are similar to the ruffs, in which the inversion genotype is lethal in the homozygous state [21], although the exact details of the lack of reproductive success of the unviable morph are achieved differently than in our model. Other empirical scenarios of morph reproductive success exist, such as pure frequency dependence (i.e. without the imbalanced fitness costs among morphs with negative frequency dependence, as assumed in our model). Such negative frequency-dependent fitness can maintain three morphs, such as in side-blotched lizards [8] and the blue-tailed damselfly [67]. Nonetheless, we expect that our genomic findings—that supergenes are not required and remarkable genetic diversity can be maintained within morphs—will be robust to these differences and generally applicable. For example, the complex and variable structural rearrangements seen in *Xiphophorus birchmanni* female mimics [68] could reflect variation being maintained at causal loci, similar to what is predicted from our model. We also assumed that the loci determining the courting and parenting traits were autosomal and that each causal locus has a roughly equal, additive contribution to the trait. If the causal variants are Y-linked, such as in Para guppies [69,70], or under control of sex steroids, as might be the case in the ruff [28], then our predictions about genetic architectures might not be fully supported in these species. Altogether, although we assumed specific patterns of morph reproductive success, which might only reflect some proportion of heteromorphic species’ patterns of relative fitness, we anticipate that highly polygenic traits can persist across a wide range of empirical systems, with considerable genetic diversity being maintained, both with and without supergenes or other structural variants to group causal loci within the genome.

### (d) Summary

Here, we show that the genetic architecture of discrete morphs affects the frequency of morphs maintained under balancing selection, but does not fundamentally change the conditions enabling balancing selection to allow multiple morphs to persist. We also show surprisingly elevated genetic diversity can be maintained, but that genome-wide scans are unlikely to accurately identify the complete genetic architecture of discrete traits. Our results highlight that additively determined dichotomous traits can be polygenic and exhibit evolutionary dynamics equivalent to a single-locus model, regardless of the physical configuration of the causal variants.

## Data Availability

All code is archived on Zenodo [71]. Outputs used in the creation of figures in the main text and the supplement are archived on zenodo [72]. Supplementary material is available online [73].
